# Lived experiences in action: Relations between community health workers’ and clients’ perinatal health behaviours in India

**DOI:** 10.1080/17441692.2025.2537697

**Published:** 2025-07-25

**Authors:** Faiz A. Hashmi, Oskar Burger, Sudipta Mondal, Cristine H. Legare

**Affiliations:** a Center for Applied Cognitive Science, The University of Texas at Austin, Austin, TX, USA; b OMNI Institute, Denver, CO, USA; c PCI India, New Delhi, India

**Keywords:** Social behavioural change, Accredited Social Health Activist (ASHA), maternal and child health, traditional medicine (or health), cultural ecologies of health

## Abstract

Community health workers are crucial bridges between health systems and local populations, uniquely positioned at the intersection of personal experiences and professional duties. Yet, limited research examines how these workers’ personal health decisions influence their clients’ behaviours. This study addresses this gap by investigating associations between the maternal experiences of Accredited Social Health Activists (ASHAs) and perinatal health practices among their clients (recent mothers) in Bihar, India. Using a large-scale cross-sectional survey involving 400 ASHAs and 1166 clients, we conducted mixed-effects logistic regression analyses to examine these relationships. Our findings reveal a strong association between ASHAs’ personal practices and their clients’ behaviours during pregnancy. ASHAs who adhered to evidence-based health practices recommended by formal healthcare systems, such as early pregnancy registrations and antenatal check-ups, and those who engaged in traditional practices, such as concealing pregnancy or calling traditional midwives during labour, predicted similar practices among their clients. These results highlight the critical position of community health workers at the intersection of personal and professional spheres, where their lived experiences meaningfully influence their work. Public health systems should leverage community health workers’ shared sociocultural and personal experiences through targeted training, supporting their role in fostering sustainable maternal and child health improvement.

## Introduction

In the last two decades, India has witnessed a remarkable transformation in public health, particularly in Reproductive, Maternal, Neonatal, and Child Health (RMNCH). The nation has achieved a 77% reduction in maternal and infant mortality rates, signalling a shift towards recommended RMNCH behaviours that include practices such as timely antenatal care, institutional delivery, proper nutrition, and appropriate child healthcare behaviours (International Institute for Population Sciences (IIPS) & ICF, 2021; Office of the Registrar General India, 2020). Central to this transformation is a community health initiative called the Accredited Social Health Activist (ASHA) programme (Engmann et al., 2016; Krishnan, 2022; Maroju et al., 2023). ASHAs are Community Health Workers (CHWs), a vital cadre of frontline workers who bridge formal health systems and communities in resource-limited settings (Burger et al., 2022; Maes, 2015). Recognised by WHO as essential for universal health coverage and proven effective in improving maternal and child health, India’s ASHA programme stands as one of the world’s largest CHW initiatives and a model for global implementation. Enlisting over a million literate women (with minimum 8th standard education) from the community, these workers have been pivotal in broadening essential healthcare reach and significantly improving maternal and child health metrics in underserved areas (Gopalan et al., 2012; Saprii et al., 2015; Taneja et al., 2019). For example, exposure to ASHA services resulted in a 17% increase in initial antenatal care visits, a 5% increase in women completing four or more antenatal visits, and a 28% surge in institutional births (Agarwal et al., 2019).

As the ASHA programme continues to work towards improving RMNCH outcomes, it faces the enduring challenge of influencing culturally sensitive, norm-driven behaviours, such as the low uptake of temporary contraception methods (usage of condom and oral contraceptive pills are 9% and 2.1%, respectively) or nutrition-related practices (57.2% of mothers have anemia; only 11.3% of under-2s get an age-appropriate, diverse, and nutritionally adequate diet) (International Institute for Population Sciences (IIPS) & ICF, 2021). ASHAs confront the challenge of promoting health changes in India’s culturally diverse communities (Ali & Chauhan, 2020). The varying beliefs, norms, traditions, and community influences that form these ‘cultural ecologies’ demand a tailored approach to health education and behaviour uchange (Cutchin, 2007; Legare et al., 2020; Sparks, 2014). Such change must be rooted in a nuanced understanding of individual, environmental, and social dynamics, going beyond simple economic motivations or knowledge deficits (Bouton, 2014; Gifford & Nilsson, 2014).

Formal health systems must embrace a more complex, nuanced approach to intervention design. This is essential for ASHAs to transition effectively into roles that actively facilitate behavioural change (Schaaf et al., 2020; Scott et al., 2019). Current approaches often overlook the multifaceted nature of health behaviours in diverse cultural contexts. Most existing evaluations have focused on quantifiable metrics such as health knowledge dissemination, outcomes, and extrinsic incentives, neglecting individual motivations, self-efficacy, and broader socio-cultural belief and support systems that drive sustained behaviour change (Adams, 2020; Ormel et al., 2019; Schaaf et al., 2020; Smittenaar et al., 2020). This oversight leads to a narrow operational view of ASHAs. It reduces them to mere conduits for standardised health education and service delivery. Instead, they should be recognised as culturally competent agents who could foster substantial and lasting health behaviour changes.

ASHAs serve as a crucial link between formal health systems and communities, and they are ideally positioned to harness their understanding of cultural and social context to promote open dialogues and motivate their clients towards better health practices (Adams, 2020; Kok et al., 2017). As residents of the communities they serve, ASHAs facilitate open communication about health concerns that might otherwise be underreported or not discussed with healthcare professionals from outside the community (Pinto et al., 2020). ASHAs are valued in their communities for their cultural knowledge and trustworthiness (Burger et al., 2022). Their strength in empathetic communication is crucial for encouraging people to change their health behaviours. This practical and respected approach is what makes ASHAs effective in improving community health (Ingram et al., 2008; National Health Systems Resource Centre, 2011). Yet, their intermediary status often translates into an ‘inherent liminality’, anchoring them at the intersection of community life and the healthcare system (Martínez, 2015). Their liminal position places them in a unique and powerful position to influence health outcomes but also confronts them with obstacles, including personal conflicts, cultural and traditional incongruence, and a lack of acknowledgment and support from the formal health system (Bist et al., 2023; Kawade et al., 2021; Schroeder et al., 2018). Further investigation is needed to unpack the factors that contribute to the liminal position of ASHAs and how this liminality impacts their performance and motivation.

ASHAs’ shared experiences and personal attributes contribute to their unique perspective on community needs. However, there is a systemic oversight in leveraging ASHAs’ personal maternal experiences, which could enhance their impact (Saprii et al., 2015; Scott & Shanker, 2010). The ASHA programme, launched in 2005, has evolved, and many from the first cohort continue to serve without retirement, offering a distinct generational view on maternal health (Smittenaar et al., 2020). This generational gap presents challenges and opportunities, while ASHAs’ long-term engagement can provide valuable insight into evolving health practices in rural Bihar.

Understanding ASHAs’ own maternal behaviours – a fundamental personal experience directly relevant to their roles as community health workers – is essential for them to effectively serve as cultural facilitators and agents of social change within rural communities, as envisioned by the National Health Mission (Cometto et al., 2018). The unique strengths that ASHAs possess, arising from shared social and religious backgrounds with their clients, often remain underutilised in health systems primarily focused on knowledge dissemination (Hashmi et al., 2023). Such experiences are not merely anecdotal; they are essentially cultural and social determinants of health that can shape ASHAs into effective cultural facilitators within their communities (Ndima et al., 2015). Maternal experiences, when actively leveraged, may provide ASHAs with insights that resonate with the clients they counsel, fostering empathy and offering more nuanced guidance (Oberg & Frank, 2009). This shared maternal journey can lend authenticity to their roles and may enhance their effectiveness when advocating for maternal and child health practices (Cometto et al., 2018).

Personal testimonies from trusted influencers, like ASHAs, can play a pivotal role in promoting positive behaviour change. Research demonstrates the power of narrative persuasion, highlighting those stories uniquely engage audiences emotionally and cognitively (Laer et al., 2014; Graaf et al., 2012). Narrative persuasion research indicates that when individuals engage with others’ experiences through storytelling, they often undergo shifts in beliefs, attitudes, and behavioural intentions (Kane et al., 2016). This mechanism is particularly potent in community health contexts where health workers are perceived as credible within their communities (Kukucka & Kassin, 2014; Leippe, 1995). Community health workers like ASHAs can leverage personal experiences and challenges related to health practices to create relatable, emotionally resonant narratives that typically prove more effective than informational or logical approaches alone. The literature suggests audiences become more invested in narratives and their outcomes than in abstract health messaging (Kane et al., 2016). Understanding how community health workers’ dual identities – as both community members with lived experiences and as formal health representatives – influence their counselling effectiveness represents a critical but understudied dimension of community health programme implementation.

We propose that because ASHAs are members of the communities they serve, their personal life experiences – particularly those related to motherhood – may significantly influence the health behaviours of these communities. This study investigates whether ASHAs’ own maternal behaviours are associated with the perinatal practices adopted by their clients. By linking each ASHA with her clients, we aim to gain a holistic understanding of their role as cultural facilitators and agents of social change. Evidence suggests that ASHAs who consistently model recommended health practices are better positioned to encourage these behaviours within their communities (Bansal et al., 2021; Burger et al., 2022). Furthermore, their training and experiences equip them with the knowledge and understanding needed to effectively promote healthier lifestyles (Sarin & Lunsford, 2017; Varshney et al., 2022).

This study takes place in rural Bihar and aims to explore the unique relationship between ASHAs and their clients, who are primarily pregnant women and mothers. We begin by examining the similarities and differences in socio-demographic characteristics and maternal practices during pregnancy between ASHAs and their clients. Due to the selection criteria and health training that ASHAs receive, we hypothesise that they will significantly differ from their clients in both sociodemographic characteristics and maternal behaviours. Next, we investigate how being a community health worker impacts ASHAs’ adherence to recommended maternal health practices during their most recent childbirth experiences. The experience of being a community health worker is expected to positively influence ASHAs’ adherence to these recommended perinatal practices. Finally, we examine the extent to which ASHAs’ personal maternal experiences predict their clients’ uptake of perinatal behaviours. We hypothesise that the maternal experiences of ASHAs are strongly associated with their clients’ adoption of recommended perinatal behaviours, with ASHAs’ behaviours predicting both biomedically recommended practices and traditionally followed behaviours, reflecting their vital position as mediators between formal healthcare systems and community cultural practices.

## Methods

The data analysed in this paper is part of the larger initiative, Project RISE, which incorporated a mixed methodology to explore the factors affecting the motivation and influence of the ASHA in improving maternal and child health behaviours in Bihar. The project explored a wide range of factors at the levels of ASHAs, such as individuals, the women and families they serve, the formal and local health influencers, and the public health system.

Project RISE integrated multiple streams of data, including Focus Group Discussions (FGDs), Key Informant Interviews (KIIs), quantitative surveys, and vignettes to explore a wide range of individual, cultural, and social factors (health behaviours, motivation, rituals, influencers, etc.) to understand the nuances of behaviour change and ASHA’s placement in the process of behaviour change. This paper primarily used quantitative survey data for the analysis.

### Study setting

The study was conducted in Bihar, one of India’s most socioeconomically disadvantaged states with persistently underwhelming maternal and child health indicators. With approximately 93,000 ASHAs serving its predominantly rural population, Bihar faces significant health challenges characterised by high fertility rates (3.4 children per woman), low female literacy (53.3%), and traditional gender norms that restrict healthcare access (International Institute for Population Sciences (IIPS) & ICF, 2021; National Health Systems Resource Centre, 2021). To capture the state’s cultural diversity, four districts were strategically selected across Bihar’s distinct linguistic and cultural regions: Samastipur and Purnia from the more extensive Maithili-speaking Mithila region in the north and east; Gaya from the Magahi-speaking Magadh region in the south; and West Champaran from the Bhojpuri-speaking Bhojpur region in the west. This sampling approach deliberately balanced representation across Bihar’s three major linguistic zones, with greater weight given to the Maithili-speaking areas due to their larger geographical footprint (covering approximately 45% of the state’s population). Each historic-cultural region maintains distinct social practices, religious observances, and maternal-child health traditions.

### ASHA programme structure

ASHAs are women selected from local communities based on educational qualifications to ensure cultural compatibility and trust (Joseena, 2021). They undergo 23 days of initial training spread across five modules covering maternal and child health, communicable diseases, and healthcare systems (Gauba & Singh, 2021). This is supplemented by regular refresher training. Their responsibilities span multiple domains: serving as healthcare system liaisons, promoting preventive health measures, facilitating maternal-child health services, implementing programmes like Janani Suraksha Yojana, and conducting community mobilisation activities (Ghanekar, 2022). ASHAs receive performance-based incentives rather than fixed salaries, with payments linked to specific activities such as facilitating institutional deliveries or complete immunisation. The programme employs an ASHA Facilitator supervisory model providing oversight and support, though implementation inconsistencies have been documented (Sur & Puthal, 2022).

### Sampling and recruitment

The data collection went on for three months, starting from June 2019 to August 2019, and throughout Project RISE, investigators engaged and conducted interviews with participants for the quantitative study. Two blocks were randomly selected from each of the four districts – Samastipur, Purnia, Gaya and Bhojpur – and fifty Anganwadi Centers (AWCs) were selected from each of the eight selected blocks. Anganwadi Center (AWC) is a community-based centre in India that provides early childhood education, nutrition, and health services to children under the age of six and their mothers. AWCs are part of the Integrated Child Development Services (ICDS) programme, which was launched in 1975 by the Government of India. Because AWCs serve as a catchment area for ASHAs, they were selected as the primary sampling units (PSUs). From each of these 400 AWCs, based on random sampling from the ASHA registers, three ASHA clients were recruited randomly from each of these 50 AWCs. The final sample size was 400 surveys of ASHAs and 1166 surveys of ASHAs’ clients after 34 were excluded for incompleteness or ineligibility based on the recruitment criteria. The research team employed female local researchers familiar with regional dialects and customs to ensure cultural sensitivity and data accuracy.

The ASHAs in our sample had served between one and twenty years in the programme, with a median service duration of twelve years. Notably, 73% of the sample had their most recent childbirth before becoming ASHAs, while 27% experienced pregnancy and birth while serving as ASHAs (noting that maternal status is not a recruitment requirement for ASHAs, so this 27% includes both first-time mothers and those having additional children during their service). This temporal distribution allows us to examine how ASHA training and experience might influence personal health behaviours while acknowledging that most ASHAs’ direct maternal experiences predate their service. For clients, all births occurred within six months prior to data collection, providing contemporary data on maternal health practices. This recency helps minimise recall bias while capturing current trends in maternal health behaviour adoption.

### Materials

The research study examined perinatal behaviours through two distinct lenses: biomedically recommended practices and traditional customs during the perinatal period. The investigation covered fourteen key behaviours, split evenly between these two categories.

The outcome measures for this study were selected through a multi-stage process beginning with a literature review of RMNCH recommendations in India and Bihar, focusing on key biomedical practices during pregnancy and childbirth as outlined by NRHM. These encompass crucial health behaviours such as first-trimester pregnancy registration, four or more antenatal check-ups, iron supplementation, institutional delivery, timely breastfeeding initiation, and specific newborn care practices. We refined this list through consultation with local health experts from PCI India and ASHA supervisors and subsequently validated these measures through formative qualitative research with community members and healthcare providers. Table I provides a comprehensive classification of these selected perinatal behaviours, including their operational definitions and recommended responses.^1^

The traditional perinatal behaviours identified in our study were documented through qualitative research conducted across 21 villages in Samastipur and Nalanda districts. We employed 40 focus group discussions with younger and older mothers (4–7 participants each, segregated by religion) and 50 key informant interviews with diverse stakeholders (ASHAs, Anganwadi Workers, Traditional Birth Attendants, medical practitioners, and religious leaders). Experienced PCI researchers facilitated these discussions using standardised guidelines to explore participants’ health-related beliefs and practices throughout pregnancy and childbirth. This methodology revealed significant cultural practices – including pregnancy concealment, religious consultations, spatial restrictions, traditional birth attendance, dietary practices, post-birth isolation, and celebratory customs – might not be readily disclosed in standard surveys or that might be region-specific (Table I in supplementary material).

For analytical purposes, behaviours were coded binary (yes/no) responses. Some practices are recommended and thus coded positively when performed (such as institutional delivery), while others are discouraged and therefore reverse-coded in the analysis (such as applying substances to the umbilical cord and early bathing of the newborn). This coding framework allows for consistently evaluating whether participants followed recommended practices, regardless of whether the recommendation was to perform or avoid the behaviour (Table I in supplementary material).

The study gathered data through self-reported responses, relying on participants’ personal accounts of their experiences, beliefs, and actions. Local health workers, particularly ASHAs, often provided guidance on both biomedical and traditional practices, highlighting the coexistence of modern healthcare recommendations with cultural traditions.

### Statistical analysis

All analyses comparing the behaviours of ASHAs with their clients were conducted using a mixed-effects logistic regression approach. This method was chosen over traditional multivariate binary logistic regression to account for the hierarchical structure of our data, where client mothers are clustered within the service areas of individual ASHAs, and ASHAs are nested within Anganwadi Centers (AWCs). The mixed-effects model allowed us to estimate fixed effects (e.g. ASHA’s perinatal practices, socio-demographic characteristics) and random effects (e.g. variation across AWCs). The inclusion of Anganwadi Centers (AWCs) as random intercepts specifically addressed the non-independence of observations within these clusters, effectively accounting for repeated measures and unobserved heterogeneity at the AWC level. This strengthens the validity of our standard errors and inferences by preventing underestimation of variance due to clustering, thereby improving the generalizability of our findings.

For each of the 14 focal behaviours, we conducted two models to examine the relationships between ASHA and client practices. Model 1 examined unadjusted associations between ASHA behaviours and client behaviours to establish baseline relationships. Model 2 included control variables based on theoretical relevance and previous literature, specifically incorporating ASHA experience (years), client and ASHA education levels, caste, and religion (Burger et al., 2022). Variable selection for the adjusted models was guided by established literature on factors influencing maternal health behaviours in rural India and Bihar specifically, focusing on known sociodemographic determinants and structural factors that could confound the ASHA-client relationship (Agarwal et al., 2019; International Institute for Population Sciences (IIPS) & ICF, 2021). This two-model approach allowed us to assess both the crude associations and the relationships that persist after accounting for key confounding variables.

## Results

### Sociodemographic similarities and differences between ASHAs and their clients

Client demographics, representing mothers served by ASHAs, align closely with national data from the National Family Health Survey (NFHS-5), India’s comprehensive demographic and health survey conducted in 2019–2021 (International Institute for Population Sciences (IIPS) & ICF, 2021). The data shows incremental improvements in marriage age compared to previous generations, rising from an average of 15.9 years to 17.2 years, though still falling short of the legally prescribed minimum age of 18 years and the Sustainable Development Goal target of eliminating child marriage (International Institute for Population Sciences (IIPS) & ICF, 2021). Clients’ mean age is 24 (range 18–41), while ASHAs’ average is 38, highlighting a significant generational gap that stems from ASHAs being recruited two decades ago, in contrast to clients who reflect more recent childbirths. Early marriage remains prevalent among clients, with 25% married by age 15 and over 40% by 16. The family composition differs markedly. ASHAs typically have 3–4 children, while clients often have 1–2, reflecting their respective life stages. The average age of the youngest child for ASHAs is 13.1 years, compared to just 2.2 months for their clients. Religious composition shows both groups are predominantly Hindu, with a slightly higher percentage of Muslim clients (approximately 10% more than ASHAs).

Caste distribution reveals nuanced differences between ASHAs and their clients. India’s traditional caste system divides society into hierarchical groups that continue to influence social status, economic opportunities, and health outcomes despite legal protections. Other Backward Class (OBC), an officially designated group of socially and educationally disadvantaged castes, forms the majority in both groups, mirroring Bihar’s census data (Census of India, 2011). However, a higher proportion of ASHAs’ clients (31%) belong to Scheduled Castes (formerly ‘untouchables’ who face significant historical discrimination) compared to ASHAs (17%), while more ASHAs are from the General category (higher castes). This suggests potential underrepresentation of the most marginalised groups among ASHAs, which may impact programme reach and effectiveness among these vulnerable populations.

Literacy among ASHAs’ clients aligns with NFHS-5 data for rural Bihar, with approximately half being illiterate (International Institute for Population Sciences (IIPS) & ICF, 2021). Concerningly, 48% of illiterate clients are under 24, indicating persistent educational challenges among younger women. ASHAs are generally better educated, reflecting the minimum 8th standard recruitment requirement. Wealth assessment reveals apparent disparities – ASHAs typically possess more assets and better housing, with only 17% living in less sturdy ‘Kuccha’ houses (made with clay walls) compared to 37% of clients. A composite wealth index demonstrates ASHAs’ median wealth about 0.5 standard deviations above the mean, while clients’ is 0.25 below, underscoring a substantial socioeconomic gap. Family structure analysis shows ASHAs are more likely to live in nuclear families (45%) compared to their clients (29%), suggesting differences in autonomy or decision-making related to age and social status. Clients (recent mothers) are often under the gatekeeping influence of in-laws, particularly mothers-in-law, which affects their autonomy.

**Figure 1.**
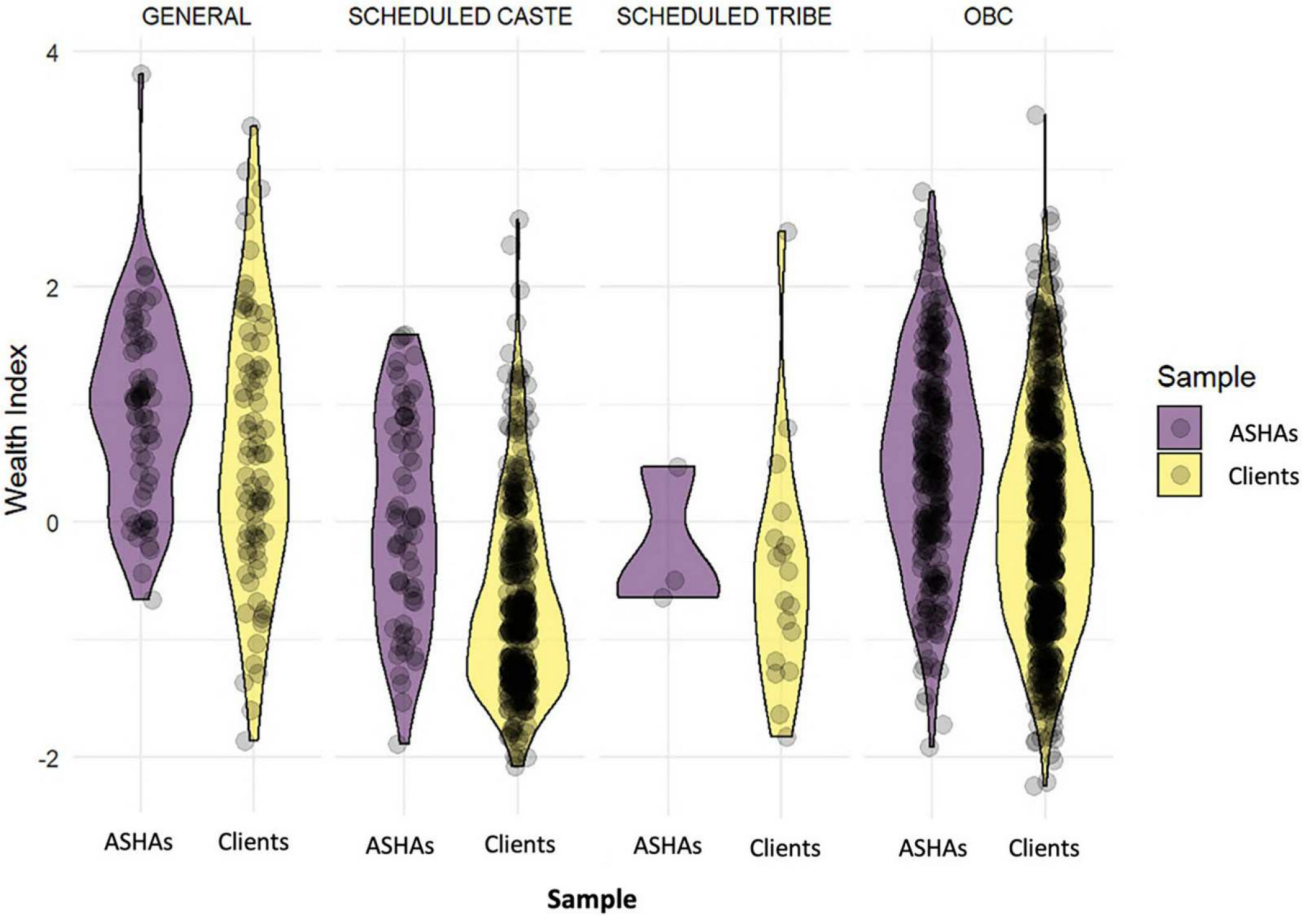
Wealth index of ASHAs and their clients (recent mothers) by caste categories. * Each row represents a separate mixed-effect regression model for each set of biomedical behaviour. The table summarises the results from these separate statistical models. See Tables XV–XXVIII in the supplementary material for complete model specifications for each traditional behaviour discussed here, including control variables (ASHA experience and caste, religion, and education for both ASHAs and their clients), random effect structures, model diagnostics, and sensitivity analyses.

Importantly, across all major caste groups, ASHAs consistently come from more affluent households (Figure 1), suggesting that socioeconomic status significantly influences ASHA selection beyond caste boundaries. While considerable variability within the ASHA group demonstrates workforce heterogeneity, this socioeconomic disparity highlights the need to address recruitment inequities to ensure more equitable representation and effective healthcare access for all community members.^2^

### Alignment of perinatal behaviours between ASHAs and their clients

We selected 14 focal behaviours practiced during the perinatal period (defined as the period from conception to the first month after delivery). The focal behaviours here are coded into biomedical (recommended or not recommended) and Traditional, and we examine variation in the responses for each sample, ASHAs, and their clients (recent mothers). This analysis introduces the idea of shared perinatal experience (ASHAs’ lived experience) and differences that exist between ASHAs and their clients in the frequency of adopting them during their most recent pregnancy.

Table 1 compares the proportions between ASHAs and their clients regarding the adoption of biomedically recommended/not recommended behaviours. These behaviours have been the priority focus of the National Rural Health Mission (NRHM) – India’s flagship health programme launched in 2005 that established the ASHA initiative – and Bihar’s state-specific health system reforms, including infrastructure improvements, strengthened supply chains for maternal health services, and community engagement (Darmstadt et al., 2020; International Institute for Population Sciences (IIPS) & ICF, 2021; Mehta et al., 2020). Some of the biomedically recommended behaviours like pregnancy registration in the first trimester (ASHA = 29%, Clients = 52%), 4 or more antenatal check-ups (ANC) (ASHA = 37.2%, Clients = 48%), and institutional delivery (ASHA = 37.2%, Clients = 82.3%) showed significantly higher levels among ASHAs’ clients, depicting a major intergeneration change from ASHAs. Similarly, some of the biomedically not recommended behaviours like applying oil /ointment on the newborn’s cord (ASHA = 68%, Clients = 58%) and bathing the child within 24 h (ASHA = 41.5%, Clients = 32.1%) were practiced by the ASHAs more than their clients. Though these differences might show an intergenerational improvement from ASHAs to their clients, they may also reflect the efforts of the health system and ASHAs as facilitators over the years.

However, the difference is not large relative to the change in biomedically recommended behaviours mentioned earlier. However, there was no difference in timely initiation of breastfeeding (ASHA = 62%, Clients = 65.2%) from ASHAs to their clients (recent mothers), and more consumption of IFA tablets (iron supplements) among ASHAs (ASHA = 45%, Clients = 34.3%) seem counterintuitive, and do not follow the intergenerational improvement we saw in other behaviours. This may reflect desirability bias but also rising resistance to IFA tablets due to reported digestive side effects and erratic supply. We need to explore further the barriers associated with these behaviours to understand the root of the problem.

We also compared ASHAs and their clients for some of the traditional behaviours that are not linked to health outcomes directly but occur during the pregnancy timeline (Table 2). The FGDs and KIIs conducted during the formative phase of project RISE showed the connectedness of these traditional behaviours with other biomedical behaviours, like the belief that avoiding going to public places during the last trimester of pregnancy may affect access to antenatal check-ups during that period. It is interesting to see minor differences in the practice of many of the traditional behaviours like celebrating Chhathi, which commemorates the arrival of the newborn into the living world (ASHA = 77.2%, Clients = 74.1%), or physical isolation of mothers and newborns in the first week after delivery (ASHA = 85.5%, Clients = 80.6%) between ASHAs and their clients, which also affirm the similar sociocultural beliefs between the two samples, but also intergenerational stability of rituals and traditions. Concealing pregnancy in the first three months (ASHA = 86.8%, Clients = 86.5%) also closely resembles both samples, which may depict the deeply rooted belief in witchcraft in the community. Pregnancy concealment in these communities stems from beliefs that early disclosure invites supernatural harm (e.g. witchcraft, evil eye), prevents social embarrassment from potentially high early miscarriage rates, and addresses limited access to timely pregnancy testing. Our previous research also identified connections between first-trimester concealment and cultural fears of the evil eye (Legare et al., 2020; Parrish et al., 2023). These similarities show how ASHAs’ beliefs mirror the community’s beliefs, reflecting shared rituals and supernatural concerns. The persistence of these behaviours suggests they are deep-seated social beliefs and norms shared by both ASHAs and the women they counsel.

### Association between ASHAs’ experience in community health and their adherence to perinatal biomedical behaviours

The primary focus of this paper is to delve into the lived experiences of ASHAs and investigate the impact of their experience on the adoption of biomedically recommended behaviours. Our sample consists of ASHAs with 1–20 years of experience, with the majority having 10–15 years. By examining ASHA’s most recent birth experiences, we assessed the extent to which their experience has influenced the adoption of health behaviours. We use two distinct models to assess the relationship between the time duration from becoming an ASHA to delivering the most recent child prior to the survey and the adoption of chosen practices. Model 1 (Table 3) scrutinises the intended health behaviour and the period as an ASHA before the previous birth, whereas Model 2 (Table 3) incorporates control factors such as age and education. Odds ratios are presented, and overall, the inclusion of age and education controls did not substantially alter the outcomes for each specific behaviour. Results show that increased experience is positively associated with key behaviours like early pregnancy registration (OR = 1.22, *p* < 0.001) and hospital deliveries (OR = 1.1, *p* < 0.001), as well as TIBF (OR = 1.06, *p* = 0.008). Conversely, more experienced ASHAs were less likely to endorse contradictory practices such as applying oil to the newborn’s cord (OR = 0.91, *p* = <0.001) and early bathing of newborns (OR = 0.96, *p* = 0.089), reflecting potential shifts towards biomedically recommended behaviours.

### Association between ASHAs’ perinatal practices and client behaviours

To assess whether ASHAs’ personal perinatal practices predict similar behaviours in their clients, we employed mixed-effects logistic models for all 14 focal behaviours (see Methods, Statistical Analysis section for detailed specifications). We examined unadjusted associations (Model 1) and associations controlling for sociodemographic factors and ASHA experience (Model 2) to isolate the specific relationship between ASHAs’ personal practices and their clients’ behaviours.

### Are ASHA’s maternal experience and practices significantly associated with the adoption of biomedical behaviours among their clients?

Our analysis of the relationship between ASHA’s biomedical perinatal behaviours and their clients’ (recent mothers) adoption of these behaviours revealed several significant associations (Table 4). In Model 1, which used ASHA’s behaviour as the sole predictor, we found that ASHAs’ clients were less likely to register their pregnancies in the first trimester if their ASHA had not done so herself (OR = 1.46, *p* = 0.02). However, ASHA’s consumption of IFA tablets, the practice of timely initiation of breastfeeding (TIBF), applying oil/ointment on the newborn’s cord, and bathing the newborn within 24 h were all associated with increased likelihood of their clients adopting these behaviours (ORs ranging from 1.27–1.33, all *p* < 0.05). Model 2, which incorporated control variables such as religion, caste, clients’ education, and ASHAs’ years of experience, showed similar trends with minor variations in the strength of associations. Notably, the association for bathing newborns within 24 h became marginally non-significant in Model 2 (OR = 1.26, *p* = 0.09). Interestingly, two behaviours (applying oil/ointment on the newborn’s cord and bathing within 24 h of birth) contradicting biomedical recommendations showed significant associations between ASHA practices and client behaviours. 4+ Antenatal Check-ups (ANCs) and institutional delivery did not show significant associations in either model. The absence of association may indicate significant intergenerational progress in antenatal care access, policy-driven incentives, and improved infrastructure for institutional check-ups and delivery services. Overall, these results suggest that ASHA’s maternal experiences and practices significantly predict the adoption of several biomedical behaviours among their clients, even after controlling for sociodemographic factors.^3^

### Are ASHA’s maternal experience and practices significantly associated with the adoption of traditional behaviours among their clients?

Our analysis of the relationship between ASHA’s traditional perinatal behaviours and their adoption among clients (recent mothers) revealed significant associations across several practices (Table 5). In Model 1, which used ASHA’s behaviour as the sole predictor, all examined traditional behaviours except ‘Consulting Priest During Pregnancy’, which showed statistically significant relationships. Notably, clients were more likely to conceal pregnancy in the first trimester (OR = 4.59, *p* < 0.001), avoid consuming cereal in the first week of childbirth (OR = 3.63, *p* < 0.001), and celebrate Chhathi on the sixth day after childbirth (OR = 12.13, *p* < 0.001) if their ASHA engaged in these practices. Furthermore, clients were less likely to avoid going to public places during pregnancy (OR = 1.40, *p* = 0.03), call a traditional birth attendant (Dai) during labour (OR = 1.98, *p* < 0.001), and practice physical isolation in the first week of childbirth (OR = 1.80, *p* = 0.02) if their ASHA did so. Model 2, which incorporated control variables including religion and caste, showed similar trends with slight variations in the strength of associations. The persistence of these relationships in both models, even after controlling for socio-demographic factors, suggests that most of ASHA’s traditional maternal practices also significantly predict adopting the same traditional behaviours among their clients.^4^ These strong associations span various aspects of perinatal care, from pregnancy concealment to postpartum practices, indicating a complex interplay between ASHAs’ personal practices and the behaviours adopted by the communities they serve.

## Discussion

Our study reveals a complex and significant relationship between ASHAs’ personal experiences, their professional roles, and their influence on community health behaviours in rural Bihar. This finding aligns with global evidence on peer-support effectiveness, defined as the capacity of individuals with similar lived experiences to positively influence each other’s health behaviours and outcomes within health systems (Dugle et al., 2023). The dual identity of ASHAs as both community members and health workers fosters a powerful peer dynamic – relationships based on shared experiences, trust, and mutual understanding – that facilitates effective health behaviour change, akin to successful community health worker programmes documented in sub-Saharan Africa and elsewhere (Downs et al., 2024). The peer dynamic observed through shared maternal experiences can help overcome barriers such as mistrust, thereby enhancing treatment adherence among marginalised populations, particularly in maternal-child health contexts (Lambert et al., 2018). We discuss our findings across three domains: socio-demographic and maternal practice differences between ASHAs and their clients, the impact of the CHW role on ASHAs’ own maternal health behaviours, and how ASHAs’ personal experiences shape their clients’ perinatal practices.

The socio-demographic profile of our sample reveals that ASHAs are, on average, older, better educated, and more economically secure than the women they counsel – attributes that simultaneously confer authority and risk widening a social-distance gap (Bianchi et al., 2025). Such asymmetries may enhance an ASHA’s credibility as a successful role model. Yet, they may also limit her ability to recognise and empathise with the structural barriers facing the most marginalised mothers. Despite this gap, we found striking concordance in both biomedical and traditional perinatal practices across the two groups, suggesting a cultural continuity that transcends socioeconomic boundaries. The high prevalence of pregnancy concealment, for instance, reflects deeply rooted beliefs about gestational vulnerability; when counsellors who should promote early antenatal care adhere to the same custom, shifting the narrative becomes difficult. Encouragingly, longitudinal studies from Uttar Pradesh show that sustained home visits allow ASHAs to bridge initial social-distance gaps, cultivate trust, and deliver effective counselling across class divides (Blanchard et al., 2021, 2024). Effective interventions must move beyond one-off knowledge transfer towards ongoing, reflective engagement: supporting ASHAs to interrogate their own practices, equipping them with culturally resonant counselling tools, and pairing behaviour-change messaging with structural supports that address the practical realities of the women they serve.

Our analysis of the impact of ASHAs’ experience on their own health behaviours yielded intriguing results. We found that longer tenure as an ASHA generally correlated with increased adoption of recommended biomedical practices, such as timely pregnancy registration and institutional delivery. This trend suggests that the ASHA role itself serves as a pathway for personal behaviour change, potentially through increased knowledge, context-aware training and support tailored to the experiences and needs of ASHAs, and a sense of responsibility as community health leaders (Agarwal et al., 2019; Hashmi et al., 2023). However, the persistence of some non-recommended practices, such as pregnancy concealment and dietary restrictions during pregnancy and postpartum, even among experienced ASHAs, indicates areas where additional training or support may be necessary.

Perhaps the most compelling findings of our study relate to the significant associations between ASHAs’ personal perinatal practices and those of their clients. For both biomedical and traditional behaviours, we observed strong associations even after controlling for sociodemographic factors, underscoring ASHAs’ powerful role as cultural facilitators whose personal experiences and choices may shape community norms and behaviours.

The varying strengths of associations across different behaviours provide valuable insights for public health interventions. Strong correlations in practices like timely initiation of breastfeeding suggest areas where ASHAs’ personal experiences could be leveraged effectively in health promotion efforts. Conversely, weaker associations for behaviours like antenatal check-ups and institutional delivery indicate that broader factors – including improved access to services and policy initiatives – have normalised these practices across generations, suggesting multi-factorial pathways of influence that extend beyond direct ASHA-client interactions and involve broader health system infrastructure, access improvements, and policy-driven changes (Jain et al., 2017; Paul & Pandey, 2020).

Our findings reveal distinct patterns requiring differentiated intervention approaches. Strong associations between ASHAs’ and clients’ traditional practices likely reflect shared community norms rather than direct ASHA influence, as both groups are embedded within the same cultural context where ASHAs navigate existing behaviours while promoting biomedical recommendations (Samuel et al., 2014). For traditional practices with high adherence that conflict with recommendations, effective interventions require community-wide approaches addressing shared cultural norms or enhanced ASHA training for sensitive navigation of traditional practices. For key recommended practices like antenatal check-ups and institutional delivery with weaker ASHA-client associations, improving outcomes requires system-level responses including strengthened health infrastructure, quality service availability, and addressing structural barriers affecting both ASHAs and clients (Legare et al., 2020).

These findings indicate that ASHA capacity-building must go beyond knowledge transfer to incorporate their lived experiences into training and supervision. Programmes should help ASHAs reflect on and selectively share personal maternity stories, boosting credibility while helping them examine norms that may conflict with biomedical advice. Given the generational gap between ASHAs and younger clients, we recommend strengthening the existing ASHA Facilitator supervisor network – successfully used in other Indian states – rather than creating new peer-to-peer schemes. This approach should include structured mentorships pairing experienced workers with new recruits, continuous updates on evolving maternal health guidance, and explicit integration of personal experiences into counselling practice, helping ASHAs navigate their dual identity as community members and health promoters.

These efforts should be complemented by formalised community feedback mechanisms – such as quarterly dialogue sessions, participatory learning cycles, or community scorecards – where ASHAs regularly engage with their clients, elders, and local leaders to discuss health challenges, share experiences and collaboratively develop contextually appropriate solutions that respectfully bridge traditional and contemporary health practices (Amosse et al., 2023). Our findings align with existing policy frameworks promoted by India’s National Health Mission (NHM), notably the communitisation processes and Participatory Learning and Action (PLA) training approaches already implemented among ASHAs (Bogler et al., 2024; Kakoti et al., 2024). While these methodologies emphasise collective community involvement, our results empirically support the rationale for leveraging ASHAs’ lived experiences to enhance these community-level interventions. This two-way communication system would enable health systems to adapt interventions based on community realities while empowering communities to influence health system approaches, creating a more responsive and culturally attuned maternal healthcare framework that builds upon established NHM foundations.

Our study underscores ASHAs’ unique position as bridges between traditional and biomedical health systems, while highlighting that their influence operates differently across practice types. For traditional practices, their embeddedness in community culture may perpetuate existing norms, requiring targeted community-wide interventions. For recommended practices, broader health system factors may outweigh individual ASHA influence, necessitating infrastructure and policy-level changes alongside enhanced ASHA support.

### Limitations

This study has several limitations, including its cross-sectional design, which precludes causal claims, and the self-reported nature of the data, which may introduce recall and social desirability biases. Additionally, while we examined key perinatal behaviours, other relevant practices may have been missed, and the temporal distance between ASHAs’ personal maternal experiences and their current roles poses limitations with potential recall bias affecting the accuracy of reported historical behaviours. Furthermore, while we controlled for several key socio-demographic and health system factors, other unmeasured variables such as household dynamics, specific community-level health promotion initiatives, or individual perceptions of ASHA quality of care could also influence client perinatal health behaviours, and the absence of data on these factors means our conclusions should be interpreted with this limitation in mind. Future research should employ longitudinal designs to explore these mechanisms over time and consider family-based approaches that capture more recent maternal experiences within ASHA households, such as those of daughters or daughters-in-law, to better understand how ASHA training influences contemporary maternal practices. Ethnographic studies could further illuminate how ASHAs navigate their dual identities and cultivate trust within cultural contexts.

## Conclusion

This study reveals significant associations between ASHAs’ personal maternal experiences and community perinatal health behaviours in rural Bihar, with distinct patterns across practice types. For traditional practices, the strong associations between ASHAs’ and clients’ behaviours likely reflect shared community norms rather than direct ASHA influence, suggesting these workers are embedded within the same cultural contexts as their clients. Conversely, for recommended practices like antenatal check-ups and institutional delivery, weaker associations indicate that broader health system factors – including infrastructure and policy initiatives – may be more influential than individual ASHA counselling.

Our findings contribute to the growing recognition that lived experience constitutes a powerful form of experiential expertise in behavioural health interventions (Paradise et al., 2010). Recognising ASHAs as cultural facilitators navigating between traditional and biomedical health systems can enhance their effectiveness, though the mechanisms differ by practice type. The persistence of traditional practices highlights the need for interventions incorporating ASHAs’ experiences within the cultural ecology of health (Betancourt et al., 2003; Whitehead, 2002).

Future interventions should empower ASHAs to reflect critically on their own beliefs and navigate the interplay between traditional and biomedical health systems (Hashmi et al., 2023; Legare et al., 2020). For traditional practices, community-wide approaches addressing shared norms may be necessary, while recommended practices may require system-level improvements in infrastructure and access. By leveraging personal narratives and lived experiences while addressing these different pathways, public health policies can design targeted strategies that strengthen community outreach efforts and address health disparities more effectively (Kane et al., 2016; Zhang et al., 2020).

## Supplementary Material

Supplementary Material

Supplemental data for this article can be accessed online at https://doi.org/10.1080/17441692.2025.2537697.

## Figures and Tables

**Table 1.** Counts and percentages of selected biomedical behaviours for ASHAs and their clients.

	ASHAs			Clients (recent mothers)		
Biomedical behaviours	*n*	%	CI	*n*	%	CI

Pregnancy Registration in First Trimester	116	29	24.65–33.76	610	52	49.14–54.94
4+ Antenatal Check-ups	149	37.2	32.53–42.21	563	48	45.15–50.94
IFA Tablet (Iron Supplement) Consumption	180	45	40.07–50.02	402	34.3	31.59–37.11
Institutional Delivery	151	37.8	33.02–42.72	965	82.3	80.01–84.46
Timely Initiation of Breastfeeding (TIBF)	248	62	57.02–66.74	764	65.2	62.37–67.90
Applying Oil/Ointment on Newborn’s Cord^a^	272	68	63.15–72.50	684	58.4	55.47–61.19
Bathing Newborn Within 24 h^a^	166	41.5	36.65–46.51	376	32.1	29.43–34.85

a These behaviours contradicted the biomedical recommendations.

**Table 2.** Counts and percentages (with confidence intervals) of selected traditional behaviours for ASHAs and their clients.

	ASHAs			Clients (recent mothers)		
Traditional behaviours	*n*	%	CI	*n*	%	CI

Concealing Pregnancy in First Trimester	347	86.8	82.9–89.8	1014	86.5	84.39–88.39
Consulting Priest During Pregnancy	29	7.2	4.99–10.36	175	14.9	12.96–17.13
Avoiding Public Places During Pregnancy	263	65.8	60.84–70.35	724	61.8	58.91–64.55
Calling Traditional Birth Attendant (Dai) During Labour	261	65.2	60.33–69.87	643	54.9	51.96–57.73
Avoiding Consuming Cereal in First Week of Childbirth^a^	149	37.2	32.53–42.21	354	30.2	27.60–32.94
Physical Isolation in First Week of Childbirth^a^	342	85.5	81.57–88.72	945	80.6	78.22–82.83
Celebrating Chhathi on Sixth Day After Childbirth	309	77.2	72.76–81.20	868	74.1	71.43–76.53

a These behaviours may have a negative effect on adjacent biomedical behaviours.

**Table 3.** The relationship between the years of experience as an ASHA and the adoption of specific biomedical behaviours is assessed as odds ratios (OR) from logistic regression analyses. Model 1 comprises solely the years as an ASHA prior to the most recent birth. Model 2 incorporates control variables such as age and education.

Biomedical behaviours of ASHAs	Model 1 odds ratio	Model 1 *P* value	Model 2 odds ratio	Model 2 *P* value

Pregnancy Registration in First Trimester	1.26	<0.001	1.22	<0.001
IFA Tablet (Iron Supplement) Consumption	1.03	0.12	1.06	0.008
Institutional Delivery	1.16	<0.001	1.1	<0.0001
Timely Initiation of Breastfeeding (TIBF)	1.08	<0.001	1.06	0.008
Applying Oil/Ointment on Newborn’s CordaE^a^	0.89	<0.001	0.91	<0.001
Bathing Newborn within 24 h^a^	0.92	<0.001	0.96	0.089

a These behaviours contradicted with the biomedical recommendations.

**Table 4.** The relationship between ASHA’s biomedical perinatal behaviours and the adoption of those behaviours among their clients (recent mothers), assessed as odds ratios (OR) from separate logistic regression analyses^a^.

Biomedical perinatal behaviours	Model 1 odds ratio	Model 1 *P* value	Model 2 odds ratio	model 2 *p* value

Pregnancy Registration in First Trimester – Clients (Predictor: Pregnancy Registration in First Trimester – ASHA)	1.46	0.02	1.42	0.03
4+ Antenatal Check-ups – Clients (Predictor: 4+ Antenatal Check-ups – ASHA)	1.13	0.35	1.06	0.69
IFA Tablets (Iron Supplement) Consumption – Clients (Predictor: IFA Tablets Consumption – ASHA)	1.30	0.05	1.37	0.04
Institutional Delivery – Clients (Predictor: Institutional Delivery – ASHA)	0.91	0.68	0.85	0.50
Timely Initiation of Breastfeeding (TIBF) – Clients (Predictor: TIBF – ASHA)	1.31	0.04	1.39	0.03
Applying Oil/Ointment on Newborn’s Cord – Clients^b^ (Predictor: Applying Oil/Ointment on Newborn’s Cord – ASHA)	1.27	0.05	1.33	0.04
Bathing Newborn Within 24 h – Clients^b^ (Predictor: Bathing Newborn Within 24 h – ASHA)	1.33	0.04	1.27	0.08

Notes: Model 1 comprises ASHAs’ behaviour as the predictor. Model 2 incorporates control variables, including religion and caste for ASHAs and their clients, clients’ education, and years of experience as ASHAs.

a Each row represents a separate mixed-effect regression model for each set of biomedical behaviours. The table summarises the results from these separate statistical models. See Tables I–XIV in the supplementary material for complete model specifications for each biomedical behaviour discussed here, including control variables (ASHA experience and caste, religion, and education for both ASHAs and their clients), random effect structures, model diagnostics, and sensitivity analyses.

b These behaviours contradicted with the biomedical recommendations.

**Table 5.** The relationship between ASHA’s traditional perinatal behaviours and the adoption of those behaviours among their clients (recent mothers), assessed as odds ratios (OR) from separate logistic regression analyses.

Traditional perinatal behaviours	Model 1 odds ratio	Model 1 *P* value	Model 2 odds ratio	Model 2 *P* value

Concealing Pregnancy in First Trimester – Clients (Predictor: Concealing Pregnancy in First Trimester – ASHA)	4.93	<0.001	5.08	<0.001
Consulting Priest During Pregnancy – Clients (Predictor: Consulting Priest During Pregnancy – ASHA)	1.67	0.07	1.52	0.15
Avoid Going to Public Places During Pregnancy – Clients (Predictor: Avoid Going to Public Places During Pregnancy – ASHA)	1.40	0.03	1.40	0.03
Calling Traditional Birth Attendant (Dai) During Labour – Clients (Predictor: Calling Dai During Labour – ASHA)	1.98	<0.001	1.67	<0.001
Avoid Consuming Cereal in First Week of Childbirth – Clients (Predictor: Avoid Consuming Cereal in First Week of Childbirth – ASHA)	3.63	<0.001	3.20	<0.001
Physical Isolation in First Week of Childbirth – Clients (Predictor: Physical Isolation in First Week of Childbirth – ASHA)	1.80	0.02	1.68	0.05
Celebrating Chhathi on Sixth Day After Childbirth – Clients (Predictor: Celebrating Chhathi on Sixth Day After Childbirth – ASHA)	12.13	<0.001	13.16	<0.001

Notes: Model 1 comprises ASHAs’ behaviour as the predictor. Model 2 incorporates control variables, including religion and caste for ASHAs and their clients, clients’ education, and years of experience as ASHAs. Each row represents a separate mixed-effect regression model for each set of biomedical behavior. The table summarizes the results from these separate statistical models. See tables XV-XXVIII in the supplementary material for complete model specifications for each traditional behavior discussed here, including control variables (ASHA experience and caste, religion, and education for both ASHAs and their clients), random effect structures, model diagnostics, and sensitivity analyses.

## Data Availability

The dataset supporting the findings of this study is openly available in the Open Science Framework (OSF) repository; Hashmi, F. A. (2024, November 13). *Dataset_Lived Experiences in Action: Relations Between Community Health Workers’ and Clients’ Perinatal Health Behaviours in India*, Retrieved from https://osf.io/xu3s6. The dataset includes anonymized survey responses from ASHAs and their clients, excluding any personally identifiable information in accordance with ethical requirements. The data contains information about maternal behaviours, sociodemographic characteristics, and health practices. Analysis scripts used in this study are also available in the same repository. Access to additional qualitative data from FGDs and KIIs is restricted due to privacy concerns but may be available from the corresponding author upon reasonable request and subject to institutional review board approval.

## Footnotes

1. See Table I in the supplementary materials for an exhaustive classification of biomedical and traditional perinatal behaviors, including their operational definitions and recommended responses.

2. See Figures I–VI in the supplementary material for more demographic information for ASHAs and their clients.

3. See Tables II–XV in the supplementary material for complete model specifications for each biomedical behavior discussed here, including control variables (ASHA experience and caste, religion, and education for both ASHAs and their clients), random effect structures, model diagnostics, and sensitivity analyses.

4. See Tables XVI–XXIX in the supplementary material for complete model specifications for each traditional behavior discussed here, including control variables (ASHA experience and caste, religion, and education for both ASHAs and their clients), random effect structures, model diagnostics, and sensitivity analyses.
