# Supplementary material for: Lived experiences in action: Relations between community health workers’ and clients’ perinatal health behaviours in India
Source: Glob Public Health. Author manuscript; Available in PMC 2025 Oct 24. (PMC12552019; doi:10.1080/17441692.2025.2537697)
Supplement: Supplementary Material [file NIHMS2110131-supplement-Supplementary_Material.docx]

**Supplementary Material**

**Tables**

* Not Applicable, as traditional perinatal behaviors do not get acknowledged or recommended through the formal health system and RMNCH guidelines

**Table I**

**The list of perinatal behaviors analyzed for the paper including the recommended response and coding definition.**

| Perinatal Behaviors | Recommended Response | Definition |
| --- | --- | --- |
| Biomedical behaviors | | |
| Pregnancy registration in First Trimester | Yes | Yes = registered with local healthcare system within first 3 months of pregnancy |
| 4+ Antenatal Checkups (ANCs) | Yes | Yes = had 4 or more checkups, No = had 0 to 3 |
| IFA Tablet (Iron Supplement) Consumption | Yes | Yes = consumed the full recommended amount: No = did not consume or consumed less than recommended |
| Hospital Delivery | Yes | Yes = government or private hospital; No = home birth |
| Timely Initiation of Breastfeeding (TIBF) | Yes | Yes = breastfed within first hour: No = after the first hour or never breastfed |
| Applying Oil/Ointment on Newborn’s Cord | No | Yes = applied something to cord stump (but there are many options for what was applied); No = applied nothing |
| Bathing Newborn within 24 Hours | No | Yes = gave bath within 24 hours; No = within 24 to 48 hrs., after 48, and bath not given |
| Traditional behaviors | | |
| Concealing Pregnancy in the First Trimester | NA* | Yes = concealed pregnancy three months or longer than three months; No = did not conceal or conceal less than two months |
| Consulting Priest During Pregnancy | NA* | Yes = consulted priest during the perinatal period; No = never consulted priest during the perinatal period |
| Avoiding Public Places During Pregnancy | NA* | Yes = avoided going to market/public places during the third trimester; No = did not avoid going to market/public places during the third trimester |
| Calling Traditional Birth Attendant (Dai) During Labor | NA* | Yes = called Dai during the labor for the last birth; No = did not call Dai during the labor for the last birth |
| Avoiding Consuming Cereal in First Week of Childbirth | NA* | Yes = Women avoided cereal-based diet during the first 6 days after last childbirth; No = Women did not avoid cereal-based diet during the first 6 days after last childbirth |
| Physical Isolation in First Week of Childbirth | NA* | Yes = Woman and newborn isolated in the first 6 days after last childbirth; No = Woman and newborn did isolate in the first 6 days after last childbirth |
| Celebrating Chhathi on Sixth Day After Childbirth | NA* | Yes = Family celebrated Chhathi on sixth day after the birth of the last child; No = Family did not celebrate Chhathi after the birth of last child |

**Table II**

**The relationship between ASHA's pregnancy registration in the first trimester and the adoption of the same behavior among their clients (recent mothers).**

| Predictors | Odds Ratios | CI | P value |
| --- | --- | --- | --- |
| Intercept | 0.99 | 0.84 – 1.16 | 0.86 |
| Pregnancy registration in First Trimester: ASHAs | 1.46 | 1.07 – 1.98 | 0.02 |

**Random Effects**

| σ² | 3.29 |
| --- | --- |
| τ₀₀ AWCreal | 0.44 |
| ICC | 0.12 |
| N AWCreal | 399 |
| Observations: | 1167 |
| Marginal R² / Conditional R²: | 0.092 / 0.127 |

**Table III**

**The relationship between ASHA’s pregnancy registration in the first trimester and the adoption of the same behavior among their clients (recent mothers). The model controls religion and caste for ASHAs and clients, clients’ education, and years of experience as ASHAs.**

| Predictors | Odds Ratios | CI | P value |
| --- | --- | --- | --- |
| Intercept | 1.26 | 0.43 – 3.70 | 0.68 |
| Client’s caste: Schedule Caste or Tribe | 1.36 | 0.76 – 2.44 | 0.30 |
| Client’s caste: OBC | 1.18 | 0.68 – 2.04 | 0.55 |
| Client’s Age | 0.97 | 0.94 – 1.00 | 0.03 |
| Client’s Religion: Muslim & Others | 0.93 | 0.60 – 1.45 | 0.75 |
| Client’s Education | 1.05 | 1.02 – 1.08 | <0.0001 |
| ASHA’s Religion: Muslim & Others | 1.69 | 0.74 – 3.87 | 0.22 |
| ASHA’s caste: Schedule Caste or Tribe | 1.05 | 0.64 – 1.74 | 0.84 |
| ASHA’s caste: OBC | 1.21 | 0.80 – 1.81 | 0.37 |
| Years as ASHA | 1.00 | 0.97 – 1.04 | 0.88 |
| Pregnancy registration in First Trimester: ASHAs | **1.42** | **1.03 – 1.95** | **0.03** |

**Random Effects**

| σ² | 3.29 |
| --- | --- |
| τ₀₀ AWCreal | 0.46 |
| ICC | 0.12 |
| N AWCreal | 399 |
| Observations: | 1163 |
| Marginal R² / Conditional R²: | 0.081 / 0.162 |

**Table IV**

**The relationship between ASHA getting 4 or more antenatal checkups (ANCs) and the adoption of the same behavior among their clients (recent mothers).**

| Predictors | Odds Ratios | CI | p |
| --- | --- | --- | --- |
| Intercept | 0.87 | 0.74 – 1.03 | 0.09 |
| 4 or more Antenatal Checkups: ASHAs | 1.13 | 0.87 – 1.48 | 0.35 |

**Random Effects**

| σ² | 3.29 |
| --- | --- |
| τ₀₀ AWCreal | 0.25 |
| ˙ICC | 0.07 |
| N AWCreal | 399 |
| Observations: | 1167 |
| Marginal R² / Conditional R²: | 0.001 / 0.072 |

**Table V**

**The relationship between ASHA getting 4 or more antenatal checkups (ANCs) and the adoption of the same behavior among their clients (recent mothers). The model controls religion and caste for ASHAs and clients, clients’ education, and years of experience as ASHAs.**

| Predictors | Odds Ratios | CI | p |
| --- | --- | --- | --- |
| Intercept | 1.82 | 0.62 – 5.33 | 0.27 |
| Client's caste: Schedule Caste or Tribe | 0.62 | 0.35 – 1.11 | 0.11 |
| Client's caste: OBC | 0.79 | 0.46 – 1.36 | 0.40 |
| Client's Age | 0.98 | 0.95 – 1.02 | 0.32 |
| Client's Religion: Muslim & Others | 1.43 | 0.93 – 2.20 | 0.10 |
| Client's Education | 1.13 | 1.10 – 1.16 | <0.001 |
| ASHA's Religion: Muslim & Others | 0.94 | 0.44 – 2.03 | 0.88 |
| ASHA's caste: Schedule Caste or Tribe | 0.85 | 0.53 – 1.38 | 0.52 |
| ASHA's caste: OBC | 0.84 | 0.57 – 1.24 | 0.39 |
| Years as ASHA | 0.95 | 0.92 – 0.98 | 0.01 |
| 4 or more Antenatal Checkups: ASHAs | 1.06 | 0.80 – 1.40 | 0.69 |

**Random Effects**

| σ² | 3.29 |
| --- | --- |
| τ₀₀ AWCreal | 0.25 |
| ICC | 0.07 |
| N AWCreal | 399 |
| Observations: | 1163 |
| Marginal R² / Conditional R²: | 0.129 / 0.190 |

**Table VI**

**The relationship between ASHA consuming IFA tablets (iron supplement) during pregnancy and the adoption of the same behavior among their clients (recent mothers).**

| Predictors | Odds Ratios | CI | p |
| --- | --- | --- | --- |
| Intercept | **0.45** | **0.37 – 0.54** | **<0.001** |
| IFA Tablets (Iron Supplement) Consumption: ASHAs | **1.30** | **1.00 – 1.70** | **0.05** |

**Random Effects**

| σ² | 3.29 |
| --- | --- |
| τ₀₀ AWCreal | 0.20 |
| ICC | 0.06 |
| N AWCreal | 399 |
| Observations: | 1167 |
| Marginal R² / Conditional R²: | 0.061 / 0.097 |

**Table VII**

**The relationship between ASHA consuming IFA tablets (iron supplement) during pregnancy and the adoption of the same behavior among their clients (recent mothers). The model controls religion and caste for ASHAs and clients, clients’ education, and years of experience as ASHAs.**

| Predictors | Odds Ratios | CI | p |
| --- | --- | --- | --- |
| Intercept | 0.22 | 0.07 – 0.66 | 0.01 |
| Client's caste: Schedule Caste or Tribe | 0.51 | 0.29 – 0.90 | 0.02 |
| Client's caste: OBC | 0.57 | 0.33 – 0.96 | 0.05 |
| Client's Age | 1.01 | 0.98 – 1.04 | 0.55 |
| Client's Religion: Muslim & Others | 0.86 | 0.55 – 1.34 | 0.51 |
| Client's Education | 1.12 | 1.09 – 1.15 | <0.001 |
| ASHA's Religion: Muslim & Others | 1.39 | 0.64 – 3.00 | 0.40 |
| ASHA's caste: Schedule Caste or Tribe | 0.97 | 0.58 – 1.60 | 0.90 |
| ASHA's caste: OBC | 1.57 | 1.05 – 2.35 | 0.03 |
| Years as ASHA | 1.02 | 0.99 – 1.06 | 0.22 |
| IFA Tablets (Iron Supplement) Consumption: ASHAs | **1.37** | **1.17 – 1.66** | **0.04** |

**Random Effects**

| σ² | 3.29 |
| --- | --- |
| τ₀₀ AWCreal | 0.15 |
| ICC | 0.04 |
| N AWCreal | 399 |
| Observations: | 1163 |
| Marginal R² / Conditional R²: | 0.114 / 0.128 |

**Table VIII**

**The relationship between ASHA giving birth in a hospital and the adoption of the same behavior among their clients (recent mothers).**

| Predictors | Odds Ratios | CI | p |
| --- | --- | --- | --- |
| Intercept | 7.35 | 5.25 – 10.30 | <0.001 |
| Hospital Delivery: ASHAs | **0.91** | **0.59 – 1.40** | **0.68** |

**Random Effects**

| σ² | 3.29 |
| --- | --- |
| τ₀₀ AWCreal | 1.44 |
| ICC | 0.30 |
| N AWCreal | 399 |
| Observations: | 1167 |
| Marginal R² / Conditional R²: | 0.000 / 0.305 |

**Table IX**

**The relationship between ASHA giving birth in a hospital and the adoption of the same behavior among their clients (recent mothers). The model controls religion and caste for ASHAs and clients, clients’ education, and years of experience as ASHAs.**

| Predictors | Odds Ratios | CI | p |
| --- | --- | --- | --- |
| Intercept | 47.85 | 8.06 – 284.00 | <0.001 |
| Client's caste: Schedule Caste or Tribe | 0.15 | 0.05 – 0.49 | 0.002 |
| Client's caste: OBC | 0.25 | 0.08 – 0.80 | 0.02 |
| Client's Age | 0.97 | 0.93 – 1.01 | 0.19 |
| Client's Religion: Muslim & Others | 0.81 | 0.42 – 1.53 | 0.51 |
| Client's Education | 1.09 | 1.05 – 1.14 | <0.01 |
| ASHA's Religion: Muslim & Others | 1.98 | 0.50 – 7.93 | 0.33 |
| ASHA's caste: Schedule Caste or Tribe | 0.83 | 0.39 – 1.76 | 0.63 |
| ASHA's caste: OBC | 1.07 | 0.58 – 1.98 | 0.83 |
| Years as ASHA | 1.00 | 0.94 – 1.05 | 0.88 |
| Hospital Delivery: ASHAs | **0.85** | **0.54 – 1.35** | **0.50** |

**Random Effects**

| σ² | 3.29 |
| --- | --- |
| τ₀₀ AWCreal | 1.46 |
| ICC | 0.31 |
| N AWCreal | 399 |
| Observations: | 1163 |
| Marginal R² / Conditional R²: | 0.102 / 0.378 |

**Table X**

**The relationship between timely initiation of breastfeeding (TIBF) to newborns among ASHAs and the adoption of the same behavior among their clients (recent mothers).**

| Predictors | Odds Ratios | CI | p |
| --- | --- | --- | --- |
| Intercept | 1.67 | 1.35 – 2.07 | **<0.001** |
| Timely Initiation of Breastfeeding (TIBF): ASHAs | **1.31** | **1.08 – 1.69** | **0.04** |

**Random Effects**

| σ² | 3.29 |
| --- | --- |
| τ₀₀ AWCreal | 0.24 |
| ICC | 0.07 |
| N AWCreal | 399 |
| Observations: | 1167 |
| Marginal R² / Conditional R²: | 0.072 / 0.138 |

**Table XI**

**The relationship between timely initiation of breastfeeding (TIBF) to newborns among ASHAs and the adoption of the same behavior among their clients (recent mothers). The model controls religion and caste for ASHAs and clients, clients’ education, and years of experience as ASHAs.**

| Predictors | Odds Ratios | CI | p |
| --- | --- | --- | --- |
| Intercept | 0.52 | 0.18 – 1.51 | 0.23 |
| Client's caste: Schedule Caste or Tribe | 1.33 | 0.76 – 2.36 | 0.32 |
| Client's caste: OBC | 1.17 | 0.69 – 1.99 | 0.57 |
| Client's Age | 1.02 | 0.99 – 1.06 | 0.15 |
| Client's Religion: Muslim & Others | 0.97 | 0.64 – 1.49 | 0.90 |
| Client's Education | 1.01 | 0.99 – 1.04 | 0.31 |
| ASHA's Religion: Muslim & Others | 1.26 | 0.58 – 2.78 | 0.56 |
| ASHA's caste: Schedule Caste or Tribe | 0.85 | 0.53 – 1.37 | 0.51 |
| ASHA's caste: OBC | 1.01 | 0.68 – 1.49 | 0.96 |
| Years as ASHA | 1.04 | 1.00 – 1.08 | 0.03 |
| Timely Initiation of Breastfeeding (TIBF): ASHAs | **1.39** | **1.11 – 1.75** | **0.03** |

**Random Effects**

| σ² | 3.29 |
| --- | --- |
| τ₀₀ AWCreal | 0.23 |
| ICC | 0.07 |
| N AWCreal | 399 |
| Observations: | 1163 |
| Marginal R² / Conditional R²: | 0.078 / 0.147 |

**Table XII**

**The relationship between ASHAs applying oil/Ointment on their newborns’ cords and the adoption of the same behavior among their clients (recent mothers).**

| Predictors | Odds Ratios | CI | p |
| --- | --- | --- | --- |
| Intercept | 1.24 | 0.97 – 1.58 | 0.082 |
| Applying Oil/Ointment on Newborn’s Cord: ASHAs | **1.27** | **1.05 – 1.63** | **0.05** |

**Random Effects**

| σ² | 3.29 |
| --- | --- |
| τ₀₀ AWCreal | 0.43 |
| ICC | 0.11 |
| N AWCreal | 399 |
| Observations: | 1167 |
| Marginal R² / Conditional R²: | 0.053 / 0.158 |

**Table XIII**

**The relationship between ASHAs applying oil/Ointment on their newborns’ cords and the adoption of the same behavior among their clients (recent mothers). The model controls religion and caste for ASHAs and clients, clients’ education, and years of experience as ASHAs.**

| Predictors | Odds Ratios | CI | p |
| --- | --- | --- | --- |
| Intercept | 1.13 | 0.38 – 3.37 | 0.82 |
| Client's caste: Schedule Caste or Tribe | 1.47 | 0.82 – 2.63 | 0.20 |
| Client's caste: OBC | 1.37 | 0.79 – 2.37 | 0.26 |
| Client's Age | 0.99 | 0.96 – 1.03 | 0.74 |
| Client's Religion: Muslim & Others | 1.57 | 0.99 – 2.48 | 0.05 |
| Client's Education | 0.96 | 0.93 – 0.99 | 0.01 |
| ASHA's Religion: Muslim & Others | 0.73 | 0.32 – 1.66 | 0.46 |
| ASHA's caste: Schedule Caste or Tribe | 1.46 | 0.88 – 2.43 | 0.14 |
| ASHA's caste: OBC | 1.26 | 0.84 – 1.89 | 0.27 |
| Years as ASHA | 0.98 | 0.95 – 1.02 | 0.35 |
| Applying Oil/Ointment on Newborn’s Cord: ASHAs | **1.33** | **1.15 – 1.76** | **0.04** |

**Random Effects**

| σ² | 3.29 |
| --- | --- |
| τ₀₀ AWCreal | 0.46 |
| ICC | 0.12 |
| N AWCreal | 399 |
| Observations: | 1163 |
| Marginal R² / Conditional R²: | 0.062 / 0.169 |

**Table XIV**

**The relationship between ASHA's Delayed bathing of her newborn and the adoption of the same behavior among their clients (recent mothers).**

| Predictors | Odds Ratios | CI | p |
| --- | --- | --- | --- |
| Intercept | 0.36 | 0.28 – 0.45 | **<0.001** |
| Delayed Bathing of Newborn: ASHAs | **1.33** | **1.12 – 1.85** | **0.04** |

**Random Effects**

| σ² | 3.29 |
| --- | --- |
| τ₀₀ AWCreal | 0.80 |
| ICC | 0.20 |
| N AWCreal | 392 |
| Observations: | 1142 |
| Marginal R² / Conditional R²: | 0.065 / 0.199 |

**Table XV**

**The relationship between ASHA's Delayed bathing of her newborn and the adoption of the same behavior among their clients (recent mothers). The model controls religion and years of experience for ASHAs and Clients’ caste, and religion.**

| Predictors | Odds Ratios | CI | p |
| --- | --- | --- | --- |
| Intercept | 0.25 | 0.12 – 0.54 | <0.001 |
| Client's caste: Schedule Caste or Tribe | 1.66 | 0.85 – 3.23 | 0.14 |
| Client's caste: OBC | 1.31 | 0.70 – 2.46 | 0.40 |
| Client's Religion: Muslim & Others | 1.02 | 0.62 – 1.67 | 0.95 |
| ASHA's Religion: Muslim & Others | 1.01 | 0.97 – 1.05 | 0.77 |
| Years as ASHA | 0.25 | 0.08 – 0.79 | 0.02 |
| Delayed bathing of newborn: ASHAs | **1.27** | **0.96 – 1.87** | **0.08** |

**Random Effects**

| σ² | 3.29 |
| --- | --- |
| τ₀₀ AWCreal | 0.74 |
| ICC | 0.18 |
| N AWCreal | 392 |
| Observations: | 1142 |
| Marginal R² / Conditional R²: | 0.053 / 0.206 |

**Table XVI**

**The relationship between ASHA's concealing pregnancy in first trimester and the adoption of the same behavior among their clients (recent mothers).**

| Predictors | Odds Ratios | CI | p |
| --- | --- | --- | --- |
| Intercept | 3.03 | 1.76 – 5.23 | **<0.001** |
| Concealing Pregnancy in First Trimester: ASHAs | **4.93** | **2.63 – 9.21** | **<0.001** |

**Random Effects**

| σ² | 3.29 |
| --- | --- |
| τ₀₀ AWCreal | 1.65 |
| ICC | 0.33 |
| N AWCreal | 399 |
| Observations: | 1167 |
| Marginal R² / Conditional R²: | 0.056 / 0.372 |

**Table XVII**

**The relationship between ASHA's concealing pregnancy in first trimester and the adoption of the same behavior among their clients (recent mothers). The model controls religion and caste for ASHAs and clients, clients’ education, and years of experience as ASHAs.**

| Predictors | Odds Ratios | CI | p |
| --- | --- | --- | --- |
| Intercept | 4.09 | 0.71 – 23.59 | 0.12 |
| Client's caste: Schedule Caste or Tribe | 1.84 | 0.74 – 4.54 | 0.19 |
| Client's caste: OBC | 1.27 | 0.56 – 2.91 | 0.57 |
| Client's Age | 1.01 | 0.96 – 1.06 | 0.83 |
| Client's Religion: Muslim & Others | 1.12 | 0.56 – 2.27 | 0.74 |
| Client's Education | 0.99 | 0.95 – 1.03 | 0.66 |
| ASHA's Religion: Muslim & Others | 0.42 | 0.12 – 1.48 | 0.18 |
| ASHA's caste: Schedule Caste or Tribe | 1.10 | 0.44 – 2.74 | 0.83 |
| ASHA's caste: OBC | 0.54 | 0.27 – 1.09 | 0.09 |
| Years as ASHA | 0.97 | 0.91 – 1.03 | 0.27 |
| Concealing Pregnancy in First Trimester: ASHAs | **5.08** | **2.70 – 9.54** | **<0.001** |

**Random Effects**

| σ² | 3.29 |
| --- | --- |
| τ₀₀ AWCreal | 1.53 |
| ICC | 0.32 |
| N AWCreal | 399 |
| Observations: | 1163 |
| Marginal R² / Conditional R²: | 0.092 / 0.380 |

**Table XVIII**

**The relationship between ASHA's consulting the priest during pregnancy and the adoption of the same behavior among their clients (recent mothers).**

| Predictors | Odds Ratios | CI | p |
| --- | --- | --- | --- |
| Intercept | 0.17 | 0.14 – 0.20 | **<0.001** |
| Consulting Priest During Pregnancy: ASHAs | **1.67** | **0.97 – 2.90** | **0.07** |

**Random Effects**

| σ² | 3.29 |
| --- | --- |
| τ₀₀ AWCreal | 0.00 |
| ICC | 0.31 |
| N AWCreal | 399 |
| Observations: | 1163 |
| Marginal R² / Conditional R²: | 0.005 / NA |

**Table XIX**

**The relationship between ASHA's consulting the priest during pregnancy and the adoption of the same behavior among their clients (recent mothers). The model controls religion and caste for ASHAs and clients, clients’ education, and years of experience as ASHAs.**

| Predictors | Odds Ratios | CI | p |
| --- | --- | --- | --- |
| Intercept | 0.35 | 0.09 – 1.34 | 0.13 |
| Client's caste: Schedule Caste or Tribe | 1.20 | 0.56 – 2.59 | 0.64 |
| Client's caste: OBC | 1.21 | 0.59 – 2.50 | 0.61 |
| Client's Age | 0.99 | 0.95 – 1.03 | 0.52 |
| Client's Religion: Muslim & Others | 1.21 | 0.72 – 2.02 | 0.48 |
| Client's Education | 0.96 | 0.93 – 1.00 | 0.04 |
| ASHA's Religion: Muslim & Others | 0.52 | 0.17 – 1.57 | 0.25 |
| ASHA's caste: Schedule Caste or Tribe | 1.18 | 0.66 – 2.08 | 0.58 |
| ASHA's caste: OBC | 0.94 | 0.58 – 1.52 | 0.80 |
| Years as ASHA | 0.96 | 0.92 – 0.99 | 0.03 |
| Consulting Priest During Pregnancy: ASHAs | **1.52** | **0.86 – 2.69** | **0.15** |

**Random Effects**

| σ² | 3.29 |
| --- | --- |
| τ₀₀ AWCreal | 1.53 |
| ICC | 0.32 |
| N AWCreal | 399 |
| Observations: | 1163 |
| Marginal R² / Conditional R²: | 0.092 / 0.380 |

**Table XX**

**The relationship between ASHA's avoiding going to public places during pregnancy and the adoption of the same behavior among their clients (recent mothers).**

| Predictors | Odds Ratios | CI | p |
| --- | --- | --- | --- |
| Intercept | 1.38 | 1.08 – 1.76 | **0.011** |
| Avoiding Going to Public Places During pregnancy: ASHAs | **1.40** | **1.04 – 1.90** | **0.03** |

**Random Effects**

| σ² | 3.29 |
| --- | --- |
| τ₀₀ AWCreal | 0.53 |
| ICC | 0.14 |
| N AWCreal | 399 |
| Observations: | 1163 |
| Marginal R² / Conditional R²: | 0.007 / 0.145 |

**Table XXI**

**The relationship between ASHA's avoiding going to public places during pregnancy and the adoption of the same behavior among their clients (recent mothers). The model controls religion and caste for ASHAs and clients, clients’ education, and years of experience as ASHAs.**

| Predictors | Odds Ratios | CI | p |
| --- | --- | --- | --- |
| Intercept | 5.12 | 1.66 – 15.83 | 0.01 |
| Client's caste: Schedule Caste or Tribe | 0.97 | 0.53 – 1.76 | 0.92 |
| Client's caste: OBC | 1.30 | 0.74 – 2.28 | 0.35 |
| Client's Age | 0.97 | 0.94 – 1.00 | 0.04 |
| Client's Religion: Muslim & Others | 0.66 | 0.42 – 1.04 | 0.08 |
| Client's Education | 1.02 | 0.99 – 1.05 | 0.14 |
| ASHA's Religion: Muslim & Others | 0.69 | 0.30 – 1.58 | 0.38 |
| ASHA's caste: Schedule Caste or Tribe | 0.85 | 0.50 – 1.43 | 0.54 |
| ASHA's caste: OBC | 0.84 | 0.55 – 1.28 | 0.42 |
| Years as ASHA | 0.95 | 0.91 – 0.98 | 0.01 |
| Avoiding Going to Public Places During pregnancy: ASHAs | **1.40** | **1.03 – 1.91** | **0.03** |

**Random Effects**

| σ² | 3.29 |
| --- | --- |
| τ₀₀ AWCreal | 0.52 |
| ICC | 0.14 |
| N AWCreal | 399 |
| Observations: | 1163 |
| Marginal R² / Conditional R²: | 0.040 / 0.171 |

**Table XXII**

**The relationship between ASHA's Calling Traditional Birth Attendant (Dai) during labor and the adoption of the same behavior among their clients (recent mothers).**

| Predictors | Odds Ratios | CI | p |
| --- | --- | --- | --- |
| Intercept | 0.83 | 0.62 – 1.11 | 0.199 |
| Calling Traditional Birth Attendant (Dai) During labor: ASHAs | **1.98** | **1.37 – 2.85** | **<0.001** |

**Random Effects**

| σ² | 3.29 |
| --- | --- |
| τ₀₀ AWCreal | 1.26 |
| ICC | 0.28 |
| N AWCreal | 399 |
| Observations: | 1167 |
| Marginal R² / Conditional R²: | 0.023 / 0.293 |

**Table XXIII**

**The relationship between ASHA's Calling Traditional Birth Attendant (Dai) during labor and the adoption of the same behavior among their clients (recent mothers). The model controls religion and caste for ASHAs and clients, clients’ education, and years of experience as ASHAs.**

| Predictors | Odds Ratios | CI | p |
| --- | --- | --- | --- |
| Intercept | 0.88 | 0.33 – 2.38 | 0.81 |
| Client's caste: Schedule Caste or Tribe | 4.24 | 2.46 – 7.41 | <0.001 |
| Client's caste: OBC | 2.17 | 1.31 – 3.65 | 0.003 |
| Client's Age | 0.97 | 0.94 – 1.00 | 0.03 |
| Client's Religion: Muslim & Others | 1.66 | 1.12 – 2.48 | 0.01 |
| Client's Education | 0.97 | 0.95 – 1.00 | 0.03 |
| ASHA's Religion: Muslim & Others | 0.65 | 0.32 – 1.30 | 0.22 |
| ASHA's caste: Schedule Caste or Tribe | 0.98 | 0.63 – 1.52 | 0.92 |
| ASHA's caste: OBC | 0.75 | 0.53 – 1.07 | 0.12 |
| Years as ASHA | 1.01 | 0.98 – 1.04 | 0.46 |
| Calling Traditional Birth Attendant (Dai) During labor: ASHAs | **1.67** | **1.30 – 2.15** | **<0.001** |

**Random Effects**

| σ² | 3.29 |
| --- | --- |
| τ₀₀ AWCreal | 0.52 |
| ICC | 0.14 |
| N AWCreal | 399 |
| Observations: | 1163 |
| Marginal R² / Conditional R²: | 0.040 / 0.171 |

**Table XIV**

**The relationship between ASHA's avoiding consuming cereal-based food for one week post-delivery and the adoption of the same behavior among their clients (recent mothers).**

| Predictors | Odds Ratios | CI | p |
| --- | --- | --- | --- |
| Intercept | 0.21 | 0.16 – 0.28 | **<0.001** |
| Avoiding Consuming Cereal in First Week of Childbirth: ASHAs | **3.63** | **2.52 – 5.23** | **<0.001** |

**Random Effects**

| σ² | 3.29 |
| --- | --- |
| τ₀₀ AWCreal | 0.94 |
| ICC | 0.22 |
| N AWCreal | 399 |
| Observations: | 1167 |
| Marginal R² / Conditional R²: | 0.084 / 0.288 |

**Table XXV**

**The relationship between ASHA's avoiding consuming cereal-based food for one week post-delivery and the adoption of the same behavior among their clients (recent mothers). The model controls religion and caste for ASHAs and clients, clients’ education, and years of experience as ASHAs.**

| Predictors | Odds Ratios | CI | p |
| --- | --- | --- | --- |
| Intercept | 0.51 | 0.15 – 1.72 | 0.28 |
| Client's caste: Schedule Caste or Tribe | 0.85 | 0.45 – 1.62 | 0.62 |
| Client's caste: OBC | 1.07 | 0.58 – 1.96 | 0.83 |
| Client's Age | 1.00 | 0.96 – 1.03 | 0.95 |
| Client's Religion: Muslim & Others | 0.48 | 0.28 – 0.82 | 0.01 |
| Client's Education | 1.02 | 0.99 – 1.05 | 0.15 |
| ASHA's Religion: Muslim & Others | 0.95 | 0.36 – 2.53 | 0.92 |
| ASHA's caste: Schedule Caste or Tribe | 0.72 | 0.41 – 1.27 | 0.26 |
| ASHA's caste: OBC | 0.62 | 0.40 – 0.98 | 0.04 |
| Years as ASHA | 0.96 | 0.92 – 1.00 | 0.05 |
| Avoiding Consuming Cereal in First Week of Childbirth: ASHAs | **3.20** | **2.31 – 4.43** | **<0.001** |

**Random Effects**

| σ² | 3.29 |
| --- | --- |
| τ₀₀ AWCreal | 0.74 |
| ICC | 0.18 |
| N AWCreal | 399 |
| Observations: | 1163 |
| Marginal R² / Conditional R²: | 0.098 / 0.263 |

**Table XXVI**

**The relationship between ASHA's Physical isolation in the first week of childbirth and the adoption of the same behavior among their clients (recent mothers).**

| Predictors | Odds Ratios | CI | p |
| --- | --- | --- | --- |
| Intercept | 3.41 | 2.13 – 5.45 | **<0.001** |
| Physical Isolation in First Week of Childbirth: ASHAs | **1.80** | **1.09 – 2.98** | **0.02** |

**Random Effects**

| σ² | 3.29 |
| --- | --- |
| τ₀₀ AWCreal | 0.94 |
| ICC | 0.22 |
| N AWCreal | 399 |
| Observations: | 1167 |
| Marginal R² / Conditional R²: | 0.010 / 0.230 |

**Table XXVII**

**The relationship between ASHA's Physical isolation in the first week of childbirth and the adoption of the same behavior among their clients (recent mothers). The model controls religion and caste for ASHAs and clients, clients’ education, and years of experience as ASHAs.**

| Predictors | Odds Ratios | CI | p |
| --- | --- | --- | --- |
| Intercept | 10.86 | 2.53 – 46.66 | 0.001 |
| Client's caste: Schedule Caste or Tribe | 1.12 | 0.55 – 2.29 | 0.75 |
| Client's caste: OBC | 2.35 | 1.20 – 4.59 | 0.01 |
| Client's Age | 0.96 | 0.92 – 1.00 | 0.03 |
| Client's Religion: Muslim & Others | 0.34 | 0.20 – 0.59 | <0.001 |
| Client's Education | 0.99 | 0.96 – 1.03 | 0.61 |
| ASHA's Religion: Muslim & Others | 0.74 | 0.28 – 1.99 | 0.55 |
| ASHA's caste: Schedule Caste or Tribe | 0.88 | 0.43 – 1.78 | 0.72 |
| ASHA's caste: OBC | 0.54 | 0.31 – 0.96 | 0.04 |
| Years as ASHA | 1.01 | 0.96 – 1.05 | 0.83 |
| Physical Isolation in First Week of Childbirth: ASHAs | **1.68** | **1.01 – 2.80** | **0.05** |

**Random Effects**

| σ² | 3.29 |
| --- | --- |
| τ₀₀ AWCreal | 0.88 |
| ICC | 0.21 |
| N AWCreal | 399 |
| Observations: | 1163 |
| Marginal R² / Conditional R²: | 0.074 / 0.270 |

**Table XVIII**

**The relationship between ASHA's celebrating 'Chhathi' on the sixth day after childbirth and the adoption of the same behavior among their clients (recent mothers).**

| Predictors | Odds Ratios | CI | p |
| --- | --- | --- | --- |
| Intercept | 0.69 | 0.46 – 1.04 | 0.077 |
| Celebrating Chhathi on Sixth Day After Childbirth: ASHAs | **12.13** | **6.99 – 21.06** | **<0.001** |

**Random Effects**

| σ² | 3.29 |
| --- | --- |
| τ₀₀ AWCreal | 1.82 |
| ICC | 0.36 |
| N AWCreal | 399 |
| Observations: | 1167 |
| Marginal R² / Conditional R²: | 0.175 / 0.470 |

**Table XXIX**

**The relationship between ASHA's celebrating 'Chhathi' on the sixth day after childbirth and the adoption of the same behavior among their clients (recent mothers). The model controls religion and caste for ASHAs and clients, clients’ education, and years of experience as ASHAs.**

| Predictors | Odds Ratios | CI | p |
| --- | --- | --- | --- |
| Intercept | 0.33 | 0.07 – 1.62 | 0.172 |
| Client's caste: Schedule Caste or Tribe | 1.11 | 0.50 – 2.47 | 0.792 |
| Client's caste: OBC | 1.63 | 0.77 – 3.46 | 0.202 |
| Client's Age | 1.00 | 0.96 – 1.05 | 0.934 |
| Client's Religion: Muslim & Others | 3.45 | 1.66 – 7.20 | 0.001 |
| Client's Education | 1.00 | 0.96 – 1.04 | 0.934 |
| ASHA's Religion: Muslim & Others | 1.13 | 0.31 – 4.12 | 0.855 |
| ASHA's caste: Schedule Caste or Tribe | 0.83 | 0.38 – 1.82 | 0.640 |
| ASHA's caste: OBC | 0.89 | 0.47 – 1.69 | 0.725 |
| Years as ASHA | 1.03 | 0.97 – 1.09 | 0.315 |
| Celebrating Chhathi on Sixth Day After Childbirth: ASHAs | **13.16** | **7.35 – 23.55** | **<0.001** |

**Random Effects**

| σ² | 3.29 |
| --- | --- |
| τ₀₀ AWCreal | 1.87 |
| ICC | 0.36 |
| N AWCreal | 399 |
| Observations: | 1163 |
| Marginal R² / Conditional R²: | 0.212 / 0.497 |

**Figures**

Clients


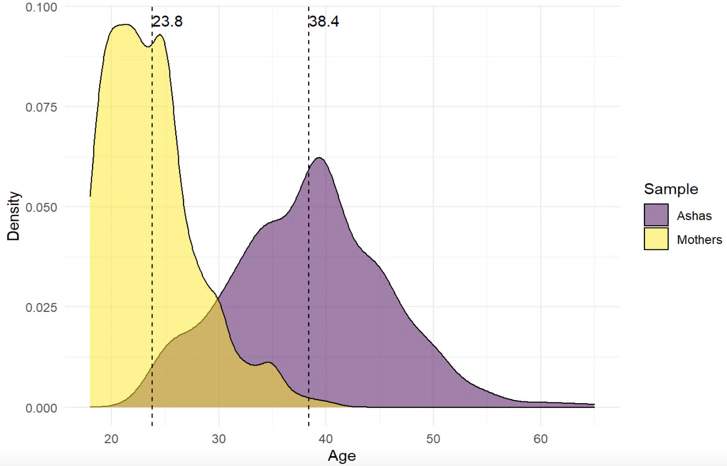


**Figure I**

**Density plot of ASHAs’ and their clients'(recent mothers) age in years**

ASHAs


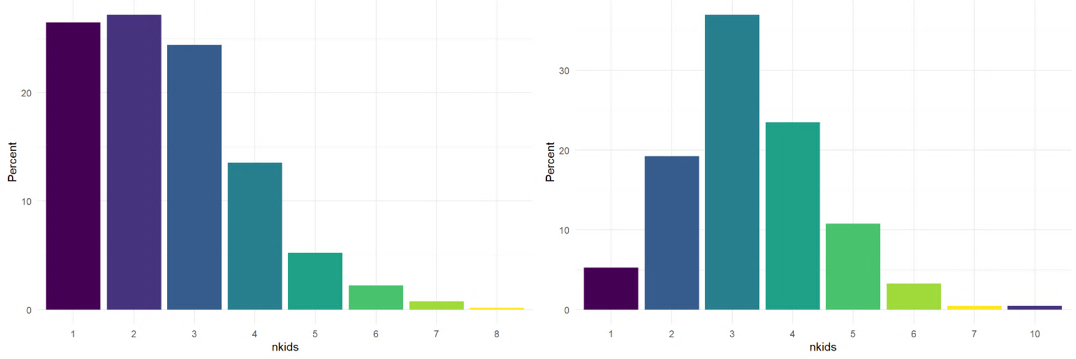


**Figure II**

**Number of living children for ASHAs (left) and their clients (right)**


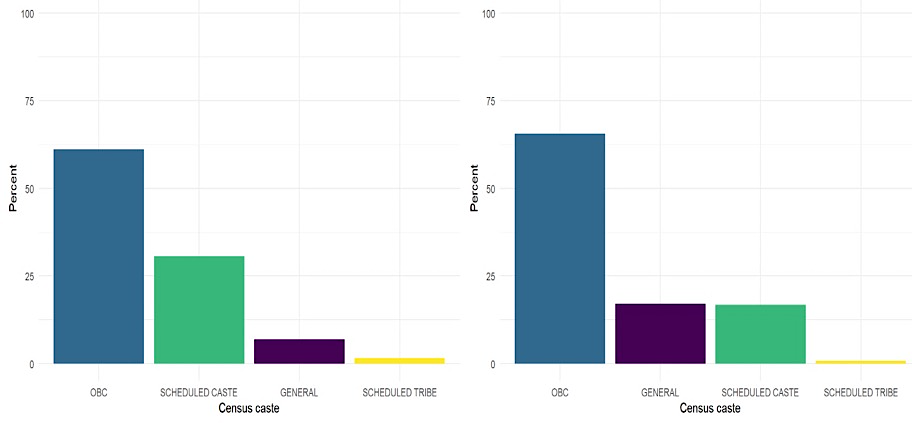


**Figure III**

**Caste distribution among ASHAs (left) and their clients (right)**

Clients

ASHAs


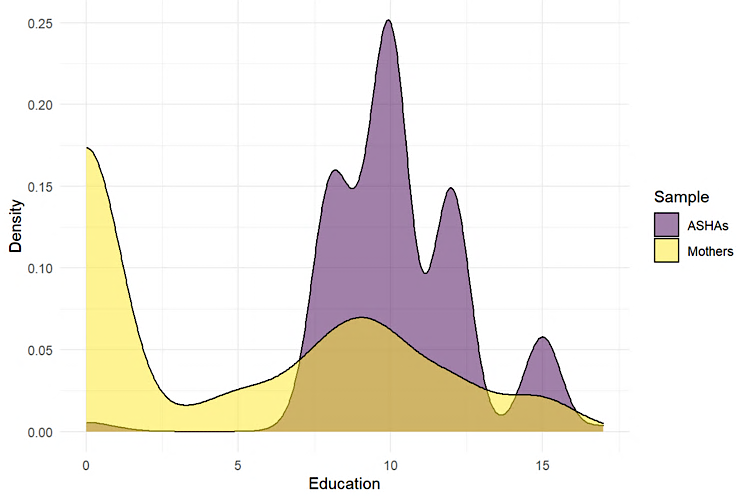


**Figure IV**

**Density plot of education among ASHAs and their clients (recent mothers)**

Clients

ASHAs


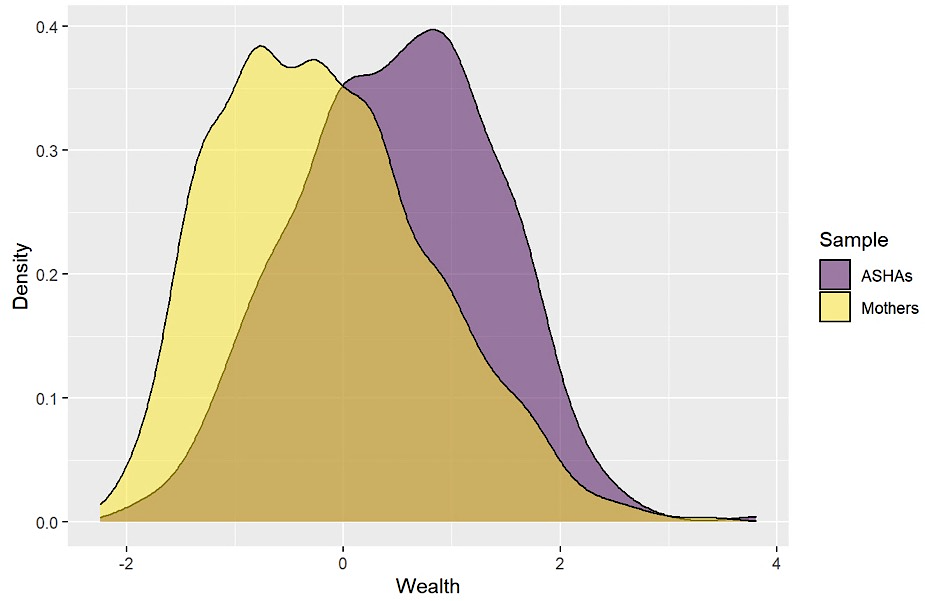


**Figure V**

**Density plot of wealth index for ASHAs and their clients (Clients)**


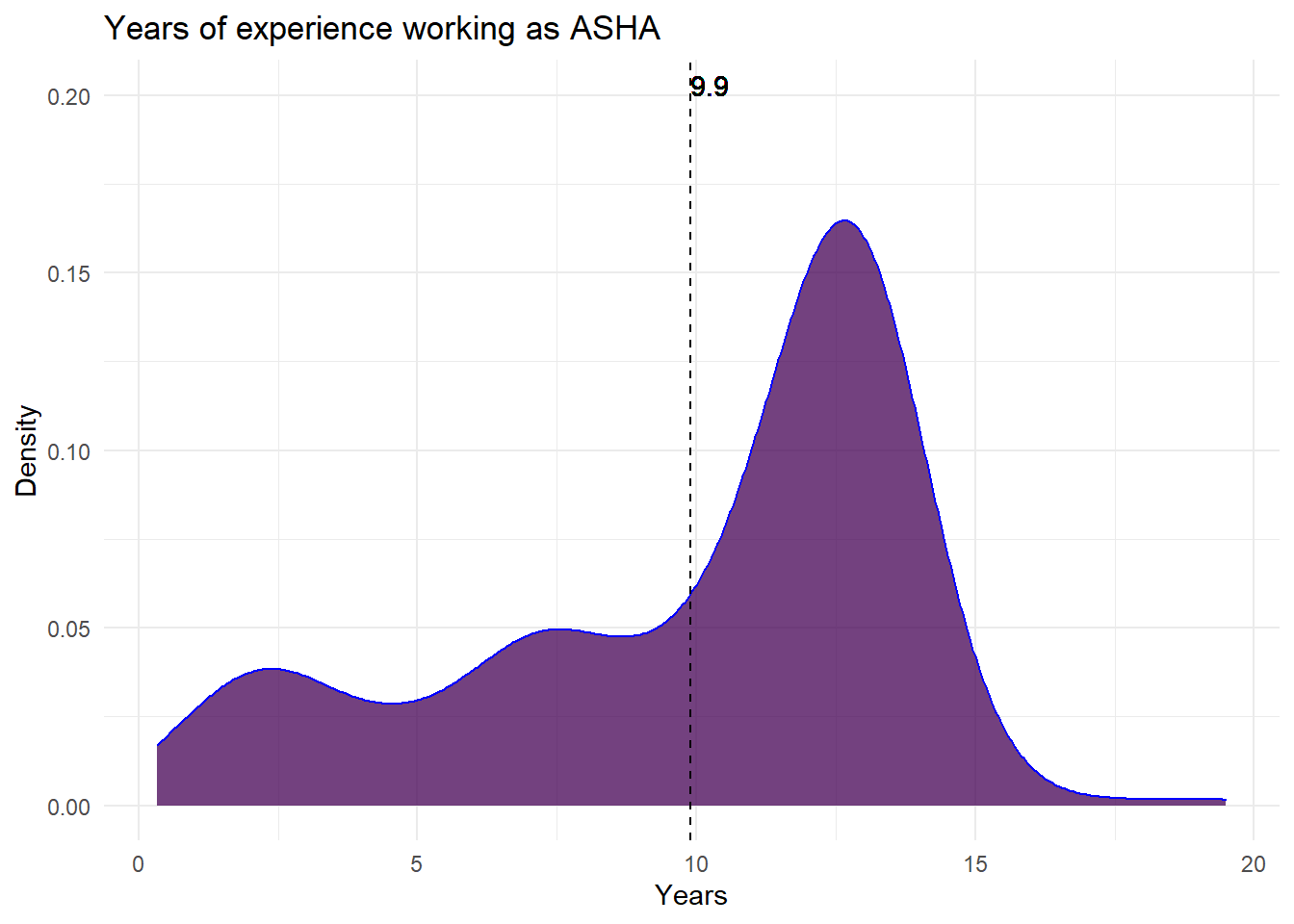


**Figure VI**

**Density plot for the years of experience working as ASHAs**
